# 5,6,7,4'-Tetramethoxyflavanone attenuates NADPH oxidase 1/4 and promotes sirtuin-1 to inhibit cell stress, senescence and apoptosis in Aß25-35–mediated SK-N-SH dysfunction

**DOI:** 10.17179/excli2021-3841

**Published:** 2021-08-23

**Authors:** Pichaya Jumnongprakhon, Ratchanaporn Chokchaisiri, Sarinthorn Thummayot, Apichart Suksamrarn, Chainarong Tocharus, Jiraporn Tocharus

**Affiliations:** 1 Department of Anatomy, Faculty of Medical Science, Naresuan University, Phitsanulok, 65000, Thailand; 2 Department of Chemistry, School of Science, University of Phayao, Phayao, 56000, Thailand; 3 Division of Anatomy, School of Medical Sciences, University of Phayao, Phayao, 56000, Thailand; 4 Department of Chemistry and Center of Excellence for Innovation in Chemistry, Faculty of Science, Ramkhamhaeng University, Bangkok 10240, Thailand; 5 Department of Anatomy, Faculty of Medicine, Chiang Mai University, Chiang Mai, 50200, Thailand; 6 Department of Physiology, Faculty of Medicine, Chiang Mai University, Chiang Mai 50200, Thailand; Center for Research and Development of Natural Products for Health, Chiang Mai University, Chiang Mai, 50200, Thailand

**Keywords:** 5,6,7,4'-Tetramethoxyflavanone, apoptosis, NOX1/4, senescence, sirtuin-1

## Abstract

Amyloidogenesis is a fundamental step of amyloid beta (Aβ) generation-induced toxicity that is commonly reported to disrupt neuronal circuits, function and survival in Alzheimer's disease (AD). The neuroprotective effect of 5,6,7,4'-tetramethoxyflavanone (TMF) from *Chormolaela odorata* extract on brain degeneration and amyloidogenesis has previously been demonstrated. However, the mechanistic evidence for TMF's effects is still unclear. In this study, we evaluated the neuroprotective effect of TMF in Aβ_25-35_-induced toxicity in SK-N-SH neuroblastoma cells. Herein, we demonstrated that TMF exhibited potent antioxidant activity and significantly increased cell viability and decreased ROS production in a dose-dependent manner. Moreover, TMF reversed the effect of Aβ_25-35_, which caused energy deprivation and apoptosis, by decreasing the ratio of Bax/Bcl-x_L_ and reducing mitochondrial membrane potential (Δψ_m_), caspase-3 expression, apoptotic cells, and attenuating glucose transporter (Glut-3) expression. In addition, TMF protected against Aβ_25-35_-induced cellular senescence by attenuating β-galactosidase, p-21 and p-53 expression and promoted the expression of Sirt-1 and p-Rb. In addition, the effects of TMF on Aβ_25-35_ toxicity were related to the upregulation of phase II antioxidant and nuclear factor erythroid 2-related factor-2 (Nrf2) signaling, including superoxide dismutase (SOD), heme oxygenase (HO)-1, and nuclear translocation of Nrf2. Finally, we also found that TMF attenuated Aβ_25-35_-reduced synaptic plasticity by increasing the expression of synaptophysin and PSD-95, which was correlated with a decrease in acetylcholine esterase (AChE). Importantly, we found that the protective effects of TMF on Aβ_25-35_ were bidirectional, including marked inhibition of NADPH oxidase (NOX)-4 activity and partial activation of Sirt-1, which occurred prior to a reduction in the negative responses. Therefore, TMF may be useful for treating Aβ toxicity in AD.

## Introduction

Alzheimer's disease (AD) has been widely reported to be the most common neurodegenerative disease and to decrease short-term memory due to the overproduction of amyloid beta (Aβ) and phosphorylated tau protein. Aβ is the major regulator of synaptic plasticity loss and neuronal cell death in the hippocampus both *in vitro* and *in vivo* (Gallardo and Holtzman, 2019[15]). The toxicity of Aβ, which induces neuronal cell death, occurs primarily by inducing energy deprivation via glucose transporter-3 (Glut-3) impairment and dysregulating the balance of Bcl-2 family proteins and mitochondrial membrane potential (Δψ_m_), which leads to caspase cascade activation and subsequent apoptosis (Chen and Zhong, 2013[7]; Leong et al., 2020[24]). Moreover, the apoptosis that occurs in response to Aβ treatment was also closely associated with several negative responses, including cellular oxidative stress, antioxidant defense imbalance and cellular senescence (Mputhia et al., 2019[30]). Previous studies have reported that Aβ mediates cellular senescence in neuronal cells by increasing β-galactosidase (β-Gal) activity, inhibiting p-retinoblastoma (p-Rb) expression and inducing p-21 and p53 expression, which are closely related to apoptosis both *in vitro* and *in vivo* (Tamagno et al., 2003[42]; Walton et al., 2020[50]). Furthermore, expression of silent mating type information regulation 2 homolog-1 (Sirt-1), which is a mediator of cellular senescence suppression and the antioxidant defense system, is also decreased in response to Aβ treatment (Ansari Dezfouli et al., 2019[2]). Meanwhile, several studies have found that the excessive levels of reactive oxygen species (ROS) produced by Aβ in neuronal tissue play a pivotal role in the induction of cellular senescence before undergoing cellular dysfunction and death. The overproduction of ROS in neuronal cells has been widely studied with respect to NADPH oxidase (NOX), especially NOX4, which is the major species of membrane-bound produced free radical in response to toxic exposure (Thummayot et al., 2018[44]). Recent studies have demonstrated that the overactivation and expression of NOX-4 are markedly related to negative responses and that inhibition of NOX4 activity reverses these effects in neuronal tissue (Oguchi et al., 2017[32]).

Generally, the cellular antioxidant defense system is activated when exposed to the stress response and then mediates and releases antioxidant enzymes to scavenge the toxin. Sirt-1 is commonly known as the gene antioxidant regulator that acts together with nuclear factor erythroid 2-related factor 2 (Nrf2) to induce the expression and nuclear translocation of the Nrf2 protein to produce phase II antioxidant enzymes, such as heme oxygenase 1 (HO-1), superoxide dismutase (SOD), and catalase (CAT) (Singh and Ubaid, 2020[38]). Several previous findings have demonstrated that excessive stress stimulation in neuronal cells by Aβ causes disturbance of the Sirt-1 and Nrf2 mechanisms, leading to progressive stress, dysfunction and death in neuronal tissue both *in vitro* and *in vivo* (Cui et al., 2018[10]; Gu et al., 2018[18]; Wang et al., 2017[51]). Finally, these negative responses induce cell death and synapse loss by decreasing the presynaptic protein (synaptophysin) and the postsynaptic protein (PSD-95) associated with an increase in acetylcholine esterase (AChE) in neuronal tissue, leading to subsequent memory loss (Chang et al., 2020[5]; Ren et al., 2020[36]).

5,6,7,4′-Tetramethoxyflavanone (TMF) is a compound extracted from *Chormolaela odorata* that is commonly known as Siam weed. The plant is commonly used in traditional medicine in Thailand for wound healing and to stop bleeding (Vijayaraghavan et al., 2017[48]). Importantly, TMF has plentiful antioxidant properties and is abundant in Thai forests (Pel et al., 2020[34]; Sirinthipaporn and Jiraungkoorskul, 2017[39]). Recently, TMF was reported to be a neuroprotective agent that reduces inflammatory responses together with amyloidogenesis prior to promoting synaptic plasticity and cognition (Pakdeepak et al., 2020[33]). Nevertheless, its effect and mechanism for alleviating Aβ toxicity in neuronal cells are not understood. Thus, we hypothesized that TMF attenuates Aβ-mediated cell stress, senescence, death, and the impairment of both the antioxidant defense system and synaptic plasticity by inhibiting NOX4 and promoting Sirt1 in SK-N-SH cells.

## Materials and Methods

### Reagents

Aβ_25-35_, N-acetylcysteine (NAC), 2,2-diphenyl-1-picrylhydrazyl (DPPH), EX527, GKT137831, and DTNB (5,5′-dithiobis-(2-nitrobenzoic acid) were purchased from Sigma-Aldrich Chemical Company (St. Louis, MO, USA). Anti-β-actin, anti-lamin B1, anti-Bcl-x_L_, anti-Bax, 3-(4,5-dimethylthiazol-2-yl)-2,5-diphenyltetrazolium bromide (MTT), 2′,7′-dichlorofluorescine diacetate, Muse® Mitopotential assay kit, Muse®Annexin V & Dead Cell kit, the Muse® Caspase-3/7 kit, and the BetaRed™ β-Gal assay kit were purchased from Merck Millipore (Millipore, MA, USA). The following antibodies were used for western blot analysis: anti-Nrf2, anti-HO-1, anti-Glut-3, anti-p21, anti-p53, anti-Rb, anti-p-Rb, anti-Sirt1, anti-synaptophysin, anti-PSD-95 (Abcam, Cambridge, UK), anti-mouse IgG peroxidase-conjugated secondary antibody, anti-rabbit IgG peroxidase-conjugated secondary antibody, goat anti-mouse IgG (rhodamine), and goat anti-rabbit IgG (Alexa Fluor® 488) (Millipore, MA, USA). The superoxide dismutase assay kit was purchased from Cayman Chemical Company (Cayman, MI, USA).

### Cell culture

Human neuroblastoma (SK-N-SH) cell lines were purchased from the American Type Culture Collection (Manassas, VA, USA) and used to assess neuronal cell properties. SK-N-SH cells were grown in minimum essential medium (MEM) supplemented with 10 % FBS, 100 U/ml penicillin, and 100 µg/ml streptomycin (GIBCO-BRL, Gaithersburg, MD) at 37 °C in a humidified 5 % CO_2_ and 95 % air incubator. The cells were cultured to 80 % confluence and passaged every 3 days by trypsinization with 0.025 % trypsin/EDTA.

### Cell viability assay

To determine the protective effect of TMF on Aβ_25-35_-induced toxicity, SK-N-SH cells at a density of 2 x 10^4^ cells/ml were seeded into 96-well microplates pretreated with various concentrations of TMF (0.1, 1, and 10 µM) for 2 hr prior to treatment with 10 µM Aβ_25-35_ for 24 hr. Thereafter, an MTT assay was performed. The absorbance was measured at 570/600 nm using a microplate reader (BioTek Instruments, Inc., USA).

### DPPH assay

The antioxidant activity of TMF was determined using a 1,1-diphenyl-2-picrylhydrazyl (DPPH) radical scavenging assay (Xie and Schaich, 2014[55]). Briefly, various concentrations of TMF (0.1, 1, and 10 µM) and a standard antioxidant, NAC (20 µM), were incubated with 100 µl of 0.3 mM DPPH solution at 30 °C for 30 min. The absorbance was measured at 517 nm using a microplate reader (BioTek Instruments, Inc., USA).

### DCFDA assay

The DCFDA assay was used to assess the production of ROS. SK-N-SH cells were cultured at a density of 2×10^4^ cells/ml in 96-well plates at 37 °C for 24 hr and exposed to EX527 (1 µM), sirt1 inhibitor or GKT137831 (1 µM), a NOX1/4 inhibitor, for 1 hr. Thereafter, cells were pretreated with various concentrations of TMF (0.1, 1, and 10 µM) for 2 hr followed by treatment with Aβ_25-35_ (10 µM) for 24 hr. H_2_DCF-DA (20 µM) was added to each well followed by incubation at 37 °C for 2 hr. Fluorescence was measured using a fluorescence microscope reader (DTX800, Beckman Coulter, Austria) at an excitation wavelength of 485 nm and an emission wavelength of 535 nm.

### Detection of apoptosis by flow cytometry

SK-N-SH cells were cultured at a density of 5×10^5^ cells/ml in 60-mm culture dishes at 37 °C for 24 hr and exposed to EX527 (1 µM) and GKT137831 (1 µM) for 1 hr. Thereafter, the cells were pretreated with TMF (10 µM) for 2 hr followed by treatment with Aβ_25-35_ (10 µM) for 24 hr. To determine the number of apoptotic cells, the cells were incubated with Muse^TM ^Annexin-V and the dead cell reaction assay kit for 20 min at room temperature. To determine caspase-3 levels, the cells were incubated with caspase-3/7 working reaction assay for 10 min at room temperature. To determine the mitochondrial potential membrane (Δψ_m_), the cells were incubated with Muse^TM ^Mito Potential working solution for 20 min at 37 °C. Finally, the number of apoptotic cells, caspase-3-positive cells, and mitochondrial potential membrane depolarization-positive cells were analyzed using a Muse Cell Analyzer (Merck Millipore, MA, USA) according to the manufacturer's instructions.

### Western blot analysis

After treatment, the cells were lysed for total and subcellular fractions of cytosolic and nuclear proteins, as previously described (Thummayot et al., 2014[45]). Protein concentrations were measured using the Bradford protein assay (BioRad, USA). The proteins in each of the fractions (50 µg) were electrophoresed in a 10 %-15 % SDS polyacrylamide gel and then transferred to a PVDF membrane (Immobilon-P, Millipore, Bedford, MA, USA). The blots were blocked for 2 hr at room temperature in 5 % skim milk in 0.1 % Tween 20 in Tris-buffered saline, pH 7.4. The membranes were incubated with primary antibodies (anti-Bcl-x_L_ (1:1000), anti-Bax (1:1000), anti-Glut-3 (1:1000), anti-p53 (1:1000), anti-p21 (1:1000), anti-p-Rb (1:1000), anti-Rb (1:1000), anti-Sirt1 (1:1000), anti-HO-1 (1:1000), anti-Nrf2 (1:1000), anti-synaptophysin (1:1000), and anti-PSD-95 (1:1000)) at 4 °C overnight. Thereafter, the membranes were probed with horseradish peroxidase-conjugated secondary antibodies (Millipore, MA, USA) for 2 hr at room temperature. The blots were incubated with Immobilon Western HRP substrate (Millipore, MA, USA). The chemiluminescent bands were detected using X-ray film. ImageJ® software (National Institutes of Health, Bethesda, MD, USA) was used for densitometry analysis. β-actin was used to normalize the total and cytosolic fractions. Lamin B1 was used to normalize the nuclear fraction.

### Senescence-associated β-galactosidase (SA-β-gal) assay

SA-β-gal was determined using a cellular senescence assay kit (Millipore, MA, USA). SK-N-SH cells at a density of 1×10^5^ cells/ml were maintained in 6-well plates at 37 °C for 24 hr. After treatment, the cells were washed twice with PBS and fixed in fixing solution for 15 min at room temperature. After washing with PBS, the cells were incubated with SA-β-Gal detection solution at 37 °C for 24 hr. Images of cells were captured under light microscopy to quantify SA-β-gal-positive cells. Experiments were performed in triplicate.

### Superoxide dismutase activity

A superoxide dismutase (SOD) assay kit (Cayman Chemical Company, MI, USA) was used to assess SOD activity in SK-N-SH cells. After the cells were treated, they were lysed in lysis buffer containing 1 % NP-40, 1 % sodium deoxycholate, 0.1 % sodium dodecyl sulfate, 40 mM β-glycerophosphate, 50 mM sodium fluoride, 2 mM sodium orthovanadate, and a cocktail of protease inhibitors. Finally, the supernatant was collected to determine SOD activity. Enzyme activity was measured at 420 nm using a microplate reader (BioTek Instruments, Inc., USA).

### Acetylcholinesterase (AChE) activity

AChE activity in SK-N-SH cells was assessed following Ellman's method (Ellman et al., 1961[13]). Briefly, the supernatant of cell lysates was mixed with High Ionic Strength (HIS) buffer containing 10 mM NaHPO_4_ 1 M NaCl, 10 % Triton X-100, and 1 mM EDTA at pH 7.0-8.0 and was incubated with Ellman's solution (3 mM DTNB in 100 mM sodium phosphate buffer, pH 7.0-8.0) at 4 °C for 20 min. Thereafter, the final concentration of 1 mM acetylthiocholine iodide was mixed in the reaction at 37 °C for 20 min. Finally, the absorbance at 405 nm was immediately read at regular intervals of 2 min using a spectrophotometer (BioTek Instruments, Inc., USA).

### Immunocytochemistry

SK-N-SH cells at a density of 1×10^3^ cells/ml were seeded into 8-well chamber slides at 37 °C for 24 hr and were then exposed to the subsequent treatments. Afterward, cells were fixed in 4 % paraformaldehyde for 20 min and permeabilized with 0.5 % Triton-X-100 for 15 min at room temperature, followed by blocking in 5 % BSA for 1 hr. Next, cells were incubated with primary antibodies (anti-synaptophysin and anti-PSD-95) in 1 % BSA at 4 °C overnight followed by incubation with goat anti-mouse IgG rhodamine-conjugated antibody and goat anti-rabbit IgG Alexa Fluor®488-conjugated antibody for 2 hr. Finally, the cells were stained with 10 µg/ml DAPI and mounted using an anti-fade reagent (Vector Laboratories, Burlingame, CA, USA). The cells were visualized under a fluorescence microscope (Olympus, IX71, Japan).

### Statistical analysis

All data are expressed as the mean ± SEM of three independent experiments. The significant difference was analyzed using one-way analysis of variance (ANOVA) followed by post hoc Dunnett's test to compare the significance between individual groups. Differences were considered significant when *p-values *< 0.05.

## Results

### TMF promotes SK-N-SH cell viability against Aβ_25-35_

**Figure 1:**
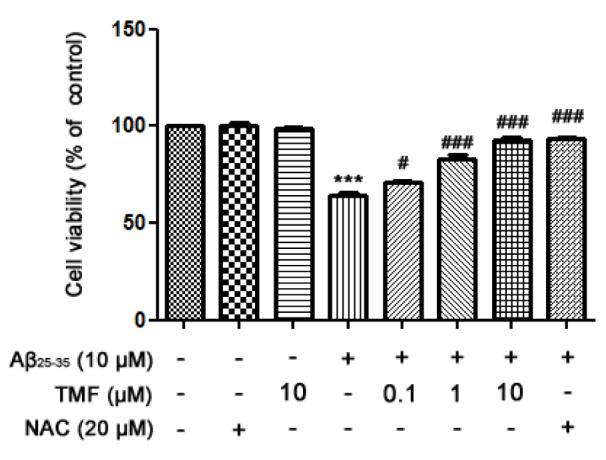
TMF promotes SK-N-SH cell viability in Aβ_25-35_-treated cells by scavenging free radicals. TMF pretreatment was analyzed using MTT assay. The values are shown as the mean ± SEM from 3 independent experiments. (** P˂0.01, *** P˂0.001, compared to the control treatment;^ #^* P*˂0.05, ^##^* P*˂0.01,^ ###^* P*˂0.001, compared to Aβ_25-35_ treatment alone).

To investigate the protective effect of TMF on Aβ_25-35_-induced neurotoxicity in SK-N-SH cells, cells were pretreated with TMF (0.1, 1, and 10 µM) for 2 hr, followed by treatment with Aβ_25-35_ for 24 hr. The results showed that TMF significantly increased cell viability in a dose-dependent manner compared to Aβ_25-35_ treatment (Figure 1(Fig. 1)). Notably, the highest dose of TMF had the high potency in the promotion of cell viability, which was similar to N-acetyl cysteine (NAC, the standard antioxidant).

### TMF ameliorates oxidative stress induced by Aβ_25-35 _in SK-N-SH cells 

**Figure 2:**
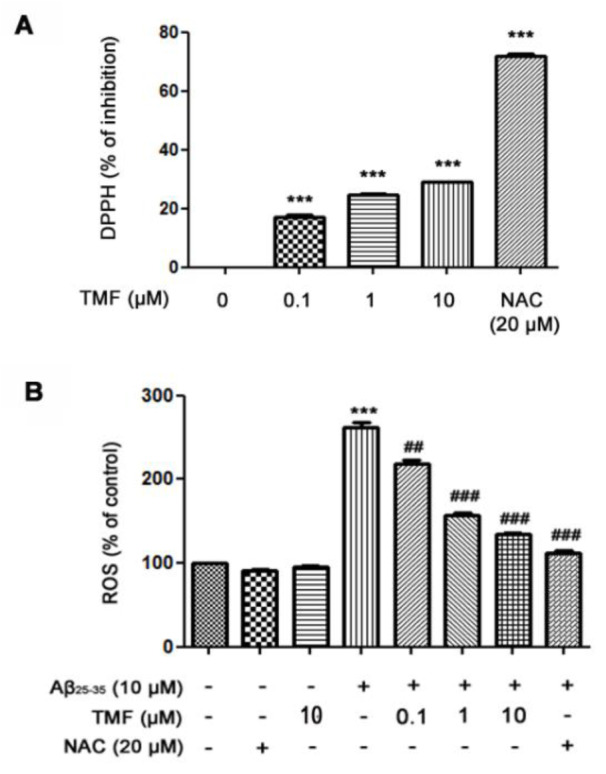
TMF scavenges extracellular free radicals and intracellular free radicals in SK-N-SH cells treated with Aβ_25-35_. A. The DPPH assay was used to determine the effect of TMF-scavenged DPPH free radicals. B. Levels of intracellular ROS production were determined using a ROS assay. The values are presented as the mean ± SEM from 3 independent experiments. (** *P*˂0.01, *** P˂0.001, compared to the control treatment; ^#^* P*˂0.05, ^##^* P*˂0.01, ^###^* P*˂0.001, compared to Aβ_25-35 _treatment alone).

To evaluate the neuroprotective activity of TMF related to antioxidant activity, we examined the radical scavenging activity of TMF using a DPPH assay. The results showed that TMF at concentrations of 0.1-10 µM significantly increased radical scavenging activity (*P *< 0.001) compared to the control group. These activities were not as strong as those of NAC, a standard antioxidant (Figure 2A(Fig. 2)). In addition, the levels of intracellular ROS were determined by measuring DCFH-DA. Our results showed that treatment with Aβ_25-35_ significantly increased ROS levels and that TMF at 0.1, 1 and 10 µM and NAC at 20 µM significantly attenuated Aβ_25-35_-induced ROS production (Figure 2B(Fig. 2)). These results suggest that TMF-improved cell viability is closely related to its antioxidant effects. TMF at a concentration of 10 µM was the most effective dose for protecting SK-N-SH cells, similar to NAC. Thus, this concentration was used in subsequent experiments.

### TMF ameliorates Aβ_25-35_-mediated apoptosis and energy deprivation

**Figure 3:**
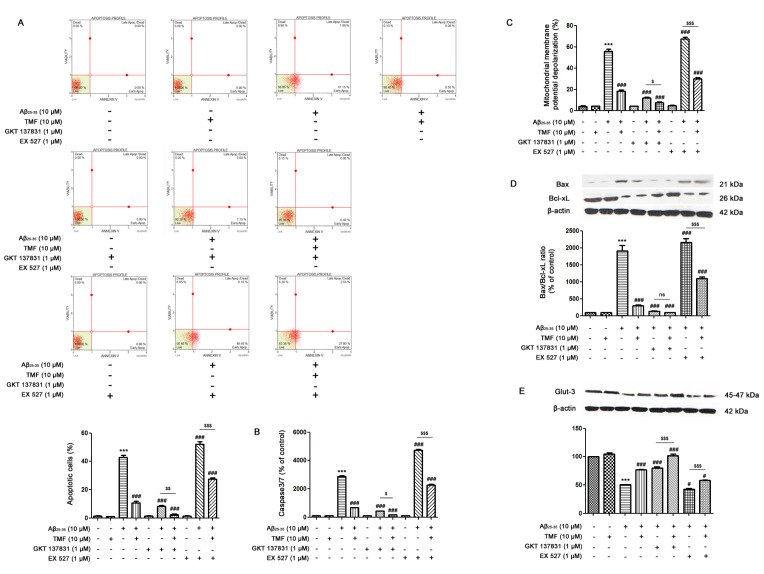
TMF prevents Aβ_25-35_-mediated apoptosis and energy deprivation via NOX1/4 inhibition and Sirt1 promotion in SK-N-SH cells. The cells were previously treated in the presence or absence of EX527 (1 µM) and GKT137831 (1 µM) for 1 hr, pretreated with TMF (10 µM) for 2 hr, and then treated with Aβ_25-35_ (10 µM) for 24 hr. Flow cytometry was used to assess A. apoptotic cells, B. caspase-3/7 levels, and C. mitochondrial membrane potential. Fifty micrograms of protein were separated using electrophoresis and analyzed by western blotting for D. the Bax/Bcl-x_L_ ratio and E. Glut-3. β-actin was used to normalize total protein levels. The values present the mean ± SEM from 3 independent experiments (*** P˂0.001, compared to the control treatment;^ #^* P*˂0.05, ^##^* P*˂0.01,^ ###^* P*˂0.001, compared to Aβ_25-35 _treatment alone; ^$^* P*˂0.05, ^$$^* P*˂0.01, ^$$$^* P*˂0.001, compared to the individual group).

Neuronal cell apoptosis induced by Aβ_25-35 _was measured by flow cytometry. The results demonstrated that Aβ_25-35_ treatment significantly increased the percentage of apoptosis (approximately 40 %) in SK-N-SH cells (*P*<0.001) (Figure 3A(Fig. 3)), which was closely associated with a significant increase in both caspase-3/7 levels (*P*<0.001) (Figure 3B(Fig. 3)) and mitochondrial membrane potential (Δψ_m_) depolarization (*P*<0.001) (Figure 3C(Fig. 3)) compared to the control group. In accordance with these results, the ratio of Bax/Bcl-x_L _expression in the Aβ_25-35_ treatment group was also significantly increased (*P* < 0.001) (Figure 3D(Fig. 3)) compared to that in the control group. Pretreatment with TMF significantly reduced caspase-3/7 levels and mitochondrial membrane potential (Δψm) depolarization, which correlated with the number of apoptotic cells. These results suggest that TMF markedly reduces apoptosis induced by Aβ_25-35_ in SK-N-SH cells. Moreover, Aβ_25-35_ promoted energy deprivation in neuronal cells by significantly decreasing the expression of GLUT-3, which is one of the major factors that caused apoptosis in SK-N-SH cells (*P*<0.001) compared to the control group (Figure 3E(Fig. 3)). In addition, pretreatment with TMF significantly increased the expression of GLUT-3. To determine whether the protective effect of TMF on Aβ_25-35_-induced apoptosis and GLUT-3 impairment was mediated by NOX1/4, cells were treated with GKT137831 (1 µM), a NOX1/4 inhibitor for 1 hr prior to pretreatment with 10 µM TMF for 2 hr, followed by 10 µM Aβ_25-35_ for 24 hr. The results revealed a decrease in Aβ_25-35_-induced apoptosis and GLUT-3 impairment in response to GKT137831 treatment compared to Aβ_25-35_ treatment alone (Figure 3A-E(Fig. 3)) (*P*<0.001). Importantly, the data demonstrated that pretreatment with TMF to inhibit NOX1/4 caused a progressive decrease in Aβ_25-_35-induced apoptosis (*P*<0.01) and GLUT-3 impairment (*P*<0.001) compared to Aβ_25-35 _alone. We further investigated the protective effect of TMF on Aβ_25-35_-induced apoptosis and GLUT-3 impairment in association with Sirt1 using EX527, a Sirt1 inhibitor. The results showed that Aβ_25-35_ treatment with EX527 significantly increased apoptosis (*P*<0.001) and GLUT-3 impairment (*P*<0.05) compared to Aβ_25-35 _treatment alone. Pretreatment with EX527 and TMF progressively decreased apoptosis and GLUT-3 impairment. However, EX527 treatment alone did not exert any effects in the cells. According to these results, we hypothesized that Aβ_25-35_-induced apoptosis and GLUT-3 impairment were closely involved in NOX1/4 activation and Sirt1 dysregulation. In addition, we also postulated that the protective effect of TMF on Aβ_25-35_-induced toxicity in SK-N-SH cells was markedly inhibited via NOX1/4 and partly via a Sirt-1-associated mechanism.

### TMF attenuates Aβ_25-35_-induced SK-N-SH cellular senescence

**Figure 4:**
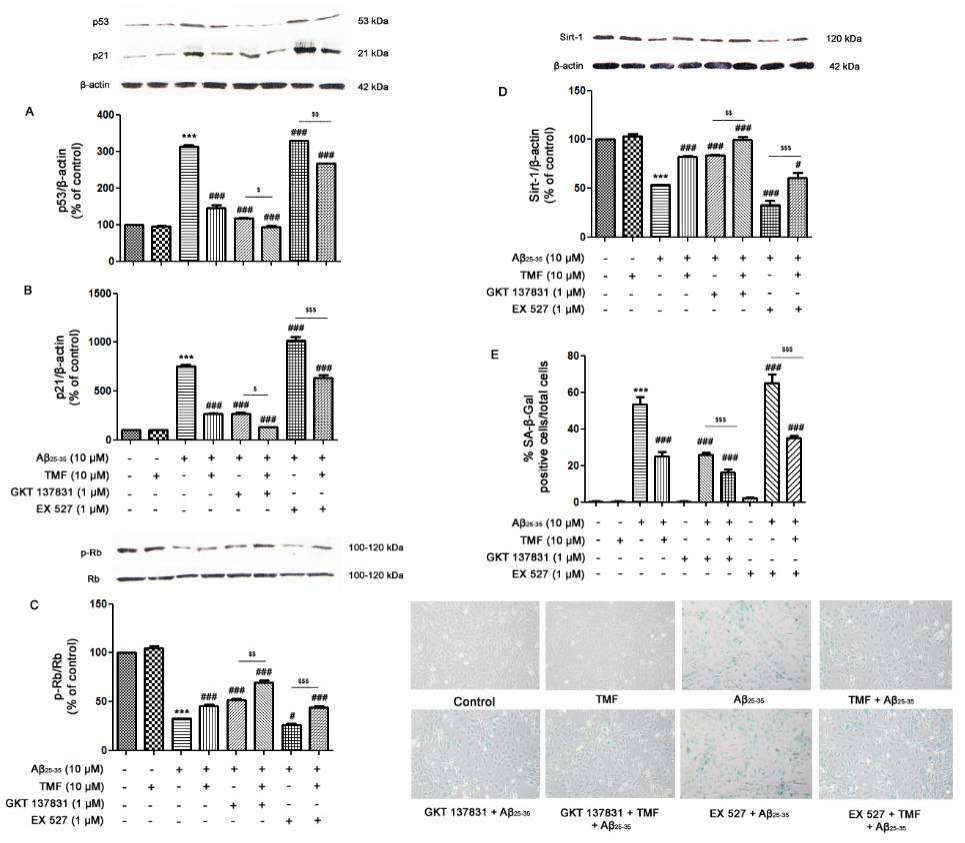
TMF protects against cellular senescence in response to Aβ_25-35 _toxicity in SK-N-SH cells via NOX1/4 and Sirt1. The cells were previously treated in the presence or absence of EX527 (1 µM) and GKT137831 (1 µM) for 1 hr, pretreated with TMF (10 µM) for 2 hr, and then treated with Aβ_25-35_ (10 µM) for 24 hr. Western blot analysis was used to determine the expression of A. p53, B. p21, C. p-Rb/Rb, and D. Sirt1. E. SA-β-galactosidase assay was used to investigate the percentage of SA-β-galactosidase-positive cells in SK-N-SH cells. The values are shown as the mean ± SEM from 3 independent experiments (*** P˂ 0.001, compared to the control treatment; ^#^* P*˂0.05, ^##^* P*˂0.01, ^###^* P*˂0.001, compared to Aβ_25-35 _treatment alone. ^$^* P*˂0.05, ^$$^* P*˂0.01, ^$$$^* P*˂0.001, compared to the individual group).

Cellular senescence is a process that is closely associated with the induction of apoptosis under both normal and stressful conditions. In accordance with previous results, we next investigated the protective role of TMF on Aβ_25-35_-induced senescence in SK-N-SH cells to determine whether it was mediated by NOX1/4 and Sirt-1. As shown in Figure 4(Fig. 4), Aβ_25-35_ significantly increased the expression of p53 (Figure 4A(Fig. 4)) and p21 (Figure 4B(Fig. 4)) and significantly decreased the expression of both p-Rb/Rb (Figure 4C(Fig. 4)) and Sirt1 (*P*<0.001) (Figure 4D(Fig. 4)) compared to the control group. TMF pretreatment significantly reduced the expression of these senescence proteins compared to Aβ_25-35_ treatment alone (*P*<0.001) (Figure 4A-E(Fig. 4)). To confirm whether TMF ameliorates Aβ_25-35_-induced senescence in SK-N-SH cells through NOX1/4, the NOX1/4 inhibitor GKT137831 was used. The results revealed that inhibition of NOX1/4 significantly reduced Aβ_25-35_-mediated cellular senescence compared to Aβ_25-35_ treatment alone (*P*<0.001), similar to TMF pretreatment (*P*<0.05) (Figure 4(Fig. 4)). Furthermore, the protective role of TMF on Aβ_25-35_-mediated cellular senescence mediated by Sirt1 was also examined. EX527 treatment caused Aβ_25-35_-induced cellular senescence compared to Aβ_25-35_ treatment alone (*P<*0.001), whereas TMF pretreatment significantly reversed these effects (*P*<0.001). These data suggest that the TMF-induced decrease in Aβ_25-35_-induced senescence markedly inhibits NOX1/4 with partial activation of Sirt-1 in SK-N-SH cells.

### TMF prevents Aβ_25-35_-suppressed Nrf2 signaling in SK-N-SH cells

**Figure 5:**
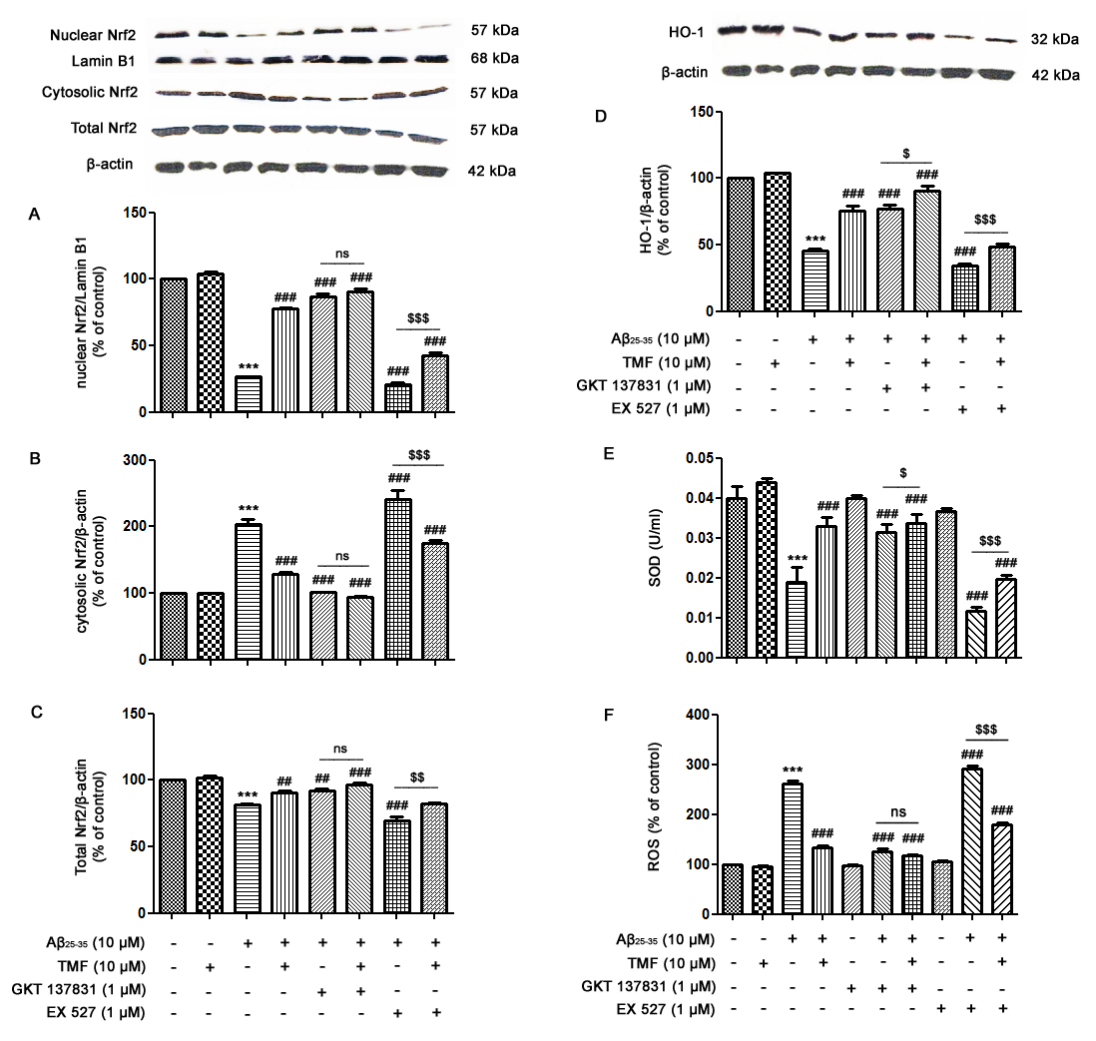
TMF attenuates Aβ_25-35_-inhibited Nrf2 signaling in SK-N-SH cells by inhibiting NOX1/4 and promoting Sirt1. The cells were previously treated in the presence or absence of EX527 (1 µM) and GKT137831 (1 µM) for 1 hr, pretreated with TMF (10 µM) for 2 hr, and then treated with Aβ_25-35_ (10 µM) for 24 hr. Equal amounts (50 µg) of protein were subjected to SDS-PAGE and analyzed by western blot analysis for the expression of A. nuclear Nrf2, B. cytosolic Nrf2, C. total Nrf2, D. HO-1 and β-actin to normalize for the cytosolic fraction and lamin B1 to normalize for the nuclear fraction. E. SOD activity was determined using a SOD assay kit. F. A ROS assay was used to determine the production of ROS in cells. The values are presented as the mean ± SEM from 3 independent experiments (*** P˂0.001, compared to the control treatment;^ #^* P*˂0.05, ^##^* P*˂0.01,^ ###^* P*˂0.001, in comparison with the Aβ_25-35 _treatment alone;^ $^* P*˂0.05, ^$$^* P*˂0.01, ^$$$^* P*˂0.001, compared to the individual group).

Nrf2 signaling is an antioxidant defense system that has been commonly accepted as a scavenger for cellular free radical-induced stress responses, including cell senescence and apoptosis. In accordance with previous results, TMF protects against Aβ_25-35_-mediated cellular senescence and apoptosis via NOX1/4 inhibition and partial Sirt1 promotion in SK-N-SH cells. We next examined the protective role of TMF in mediating antioxidant mechanisms. As shown in Figure 5(Fig. 5), Aβ_25-35 _suppressed Nrf2 signaling by significantly decreasing superoxide dismutase (SOD) activity (Figure 5E(Fig. 5)), heme oxygenase (HO)-1 (Figure 5D(Fig. 5)), total Nrf2 expression (Figure 5C(Fig. 5)), and Nrf2 translocation (Figure 5A, B(Fig. 5)) (*P*<0.001) compared to the control treatment. In contrast, TMF pretreatment reversed these effects (Figure 5(Fig. 5)) (*P*<0.001). Moreover, GKT137831 treatment significantly attenuated Nrf2-related proteins, similar to TMF pretreatment, when compared to Aβ_25-35 _treatment alone (*P*<0.001) (Figure 5A-E(Fig. 5)). In addition, NOX1/4 inhibition by GKT137831 also decreased ROS levels in both Aβ_25-35-_ and TMF-treated cells and correlated with the increase in Nrf2-related proteins when compared to Aβ_25-35 _treatment alone (*P*<0.001) (Figure 5F(Fig. 5)). Inhibition of Sirt1 by EX527 progressively suppressed Nrf2-related proteins in SK-N-SH cells treated with Aβ_25-35 _(*P*<0.001) (Figure 5(Fig. 5)) compared to Aβ_25-35 _treatment alone; however, TMF treatment restored these effects to nearly control group levels compared to Aβ_25-35 _treatment (*P*<0.001). In addition, we found that cotreatment with EX527 and TMF ameliorated Aβ_25-35_-induced suppression of Nrf2 signaling, which is closely related to ROS levels (Figure 5F(Fig. 5)). Therefore, these results suggest that TMF protects against Aβ_25-35_-suppressed Nrf2 signaling via marked NOX1/4 inhibition and partial activation of Sirt-1.

### TMF prevents synaptic plasticity in SK-N-SH cells by Aβ_25-35_

**Figure 6:**
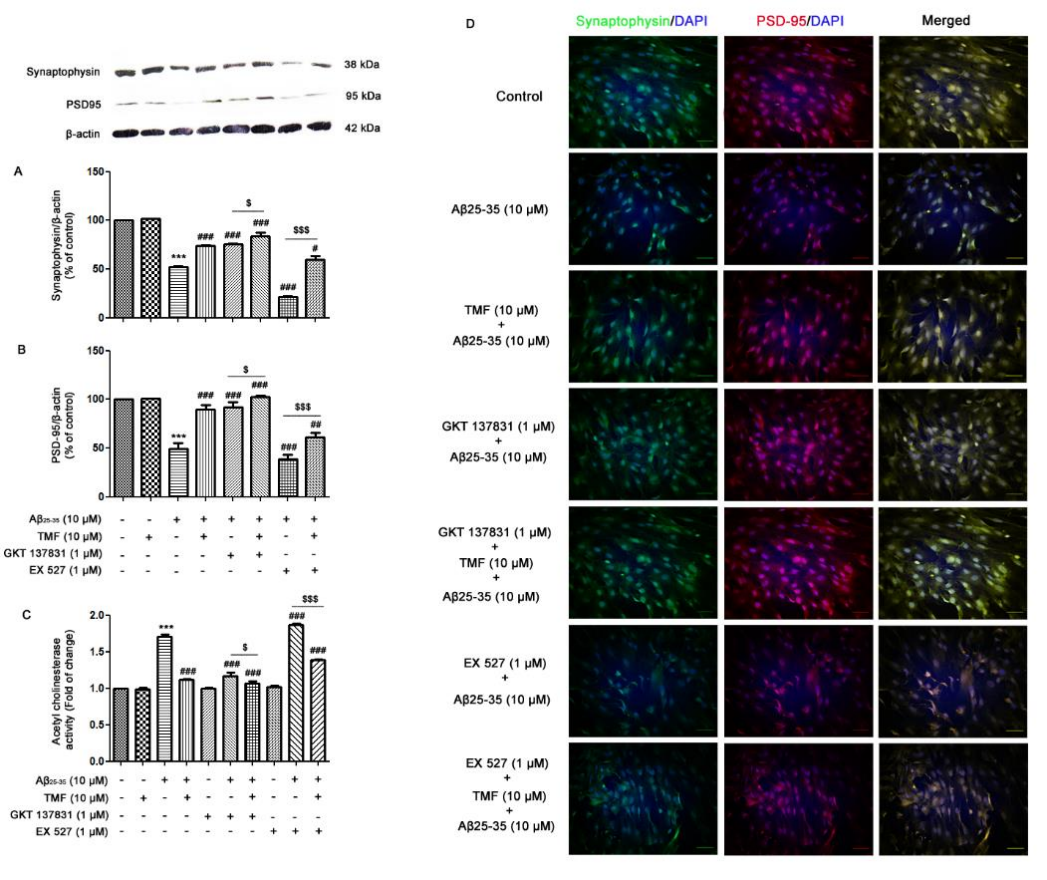
TMF reduces Aβ_25-35_-impaired synaptic plasticity via NOX1/4 and Sirt1 in SK-N-SH cells. The cells were previously treated in the presence or absence of EX527 (1 µM) and GKT137831 (1 µM) for 1 hr, pretreated with TMF (10 µM) for 2 hr, and then treated with Aβ_25-35_ (10 µM) for 24 hr. The protein expression and localization of A. synaptophysin and B. PSD-95 were determined by western blot analysis and D. immunocytochemistry. C. The activity of AChE in the cells was determined by an AChE assay. The values are presented as the mean ± SEM from 3 independent experiments (***P˂0.001, compared to the control treatment; ^#^* P<*0.05, ^##^* P*˂0.01, ^###^* P*˂0.001, compared to Aβ_25-35 _treatment alone.; ^$^* P*˂0.05, ^$$^*P*˂0.01, ^$$$^* P*˂0.001, compared to the individual group).

To determine the protective role of TMF on Aβ_25-35_-induced impairment of neuronal synaptic plasticity, we investigated whether it acts directly against NOX1/4 and Sirt1. The results demonstrated that Aβ_25-35_ induced the impairment of neuronal synapses by decreasing the expression of synaptophysin (*P*<0.001) (Figure 6A(Fig. 6)) and PSD-95 (*P*<0.001) (Figure 6B(Fig. 6)) and increasing AChE activity (*P*<0.001) (Figure 6C(Fig. 6)) compared to the control group. The alterations in synaptophysin and PSD-95 expression are shown in Figure 6D(Fig. 6) using immunofluorescence. Pretreatment with TMF prior to treatment with Aβ_25-35_ ameliorated these effects compared to Aβ_25-35 _treatment alone (*P*<0.001) (Figure 6A-D(Fig. 6)). When examining the protective effect of TMF on Aβ_25-35_-impaired neuronal synapses and whether it is involved with NOX1/4, the data demonstrated that synaptic plasticity, damaged by Aβ_25-35 _treatment, was recovered in response to GKT137831 treatment when compared to Aβ_25-35 _treatment alone (*P*<0.001), and TMF pretreatment with GKT137831 treatment progressively increased this phenomenon as well (*P*<0.001) (Figure 6A-D(Fig. 6)). Thereafter, we examined whether the role of TMF on Aβ_25-35_-induced synapse loss was related to Sirt1. The results revealed that EX527 treatment promoted the progression of synaptic plasticity impairment compared to Aβ_25-35 _treatment alone (*P*<0.001) (Figure 6A-D(Fig. 6)), while TMF pretreatment reversed these effects but was not similarly recovered to TMF pretreatment with Aβ_25-35 _treatment (*P*<0.001) (Figure 6A-D(Fig. 6)). Therefore, in this study, we suggest that the protective role of TMF on Aβ_25-35_-impaired synaptic plasticity is markedly involved with NOX1/4 and partly involved with Sirt-1 in SK-N-SH cells.

## Discussion

The generation and accumulation of Aβ peptides in the central nervous system (CNS), especially the hippocampus and cerebral cortex, has been widely considered a common cause of memory loss (Oakley et al., 2006[31]). Several *in vitro *and *in vivo* studies have demonstrated that the toxicity of Aβ_25-35_ in the CNS occurs through several mechanisms, including (1) free radical generation and cellular stress induction, (2) cellular senescence, and (3) loss of synaptic function (Diaz et al., 2012[12]; Smith and Klimov, 2018[40]). Therefore, suppressing these mechanisms could prevent neurodegeneration in AD. In the present study, we were interested in TMF, a natural flavanone that is isolated from *Chormolaela odorata. *Several lines of evidence have demonstrated that flavanone has a wide range of pharmacological and physiological effects, including anti-inflammatory, antioxidant, anti-cell death, and anticarcinogenic properties (Barreca et al., 2017[3]; Lamport et al., 2016[21]; Li and Schluesener, 2017[25]). Interestingly, our previous finding of 5,6,7,4'-tetramethoxyflavanone has high potency in the reduction of neurodegenerative pathology and amyloidogenesis in mice (Pakdeepak et al., 2020[33]). Taken together, many previous studies have demonstrated that flavanone has many pharmacological advantages, but few studies have previously mentioned its advantages with regard to TMF. However, the ability of TMF to ameliorate Aβ toxicity in neuronal cells has not yet been studied. Hence, in this study, we first investigated the underlying mechanism by which TMF attenuates neuronal cell damage mediated by cellular senescence, apoptosis, impairment of the antioxidant defense system and synaptic plasticity.

Aβ plays a pivotal role in free radical generation and apoptosis induction, and these effects are closely related to NADPH oxidase (NOX), the cell membrane-bound free radical generator (Choi et al., 2019[8]; Jiang et al., 2018[19]), and Sirt-1, the mediator for scavenging free radicals (Lee et al., 2015[23]). NOX is a membrane-bound enzyme complex that primarily produces reactive free radicals, such as superoxide anion (O_2_∙) and hydrogen peroxide (H_2_O_2_), by transferring electrons across the cell membrane and then coupling to oxygen (Tarafdar and Pula, 2018[43]). Recently, several reports have demonstrated that Aβ_25-35 _causes a significant increase in the expression of NOX1 and in neuron-generated free radicals before undergoing neuropathology (Chay et al., 2017[6]; Choi et al., 2014[9]). Our study demonstrated that TMF improved SK-N-SH cell viability in response to Aβ_25-35 _treatment and that the protective effect of TMF on Aβ_25-35 _was closely correlated with a decrease in intracellular ROS production and an increase in scavenging activity, similar to NAC. In addition, the antiapoptotic effect of TMF was also elucidated in response to Aβ_25-35 _treatment, and TMF notably mitigated apoptosis by decreasing apoptotic cells, caspase-3 expression levels, mitochondrial membrane potential polarization and the ratio of Bax/Bcl-X_L_ expression. Importantly, previous reports have demonstrated that Aβ_25-35_-induced apoptosis in neuronal cells was mediated by a GLUT3-dependent mechanism (Prapong et al., 2002[35]; Wu et al., 2019[54]). Our study also found that an increase in apoptosis was closely associated with a decrease in GLUT3 expression. TMF treatment also attenuated GLUT3 impairment in Aβ_25-35_-induced SK-N-SH cells, whereas TMF treatment alone did not show any effects. Moreover, a NOX1/4 inhibitor was found to exert the same effect as TMF on apoptosis and GLUT3 impairment induced by Aβ_25-35_. Therefore, this result suggests that apoptosis and GLUT3 impairment in response to Aβ_25-35 _is modulated by NOX1/4. However, our results also showed that TMF may act through other mechanisms by demonstrating a progressive decrease in the percentage of cell death and GLUT3 impairment observed after cotreatment with TMF and a NOX1/4 inhibitor. Next, we examined whether the protective role of TMF involved Sirt1 in response to Aβ_25-35 _treatment. Sirt-1 has also been documented to target Aβ_25-35_-induced cell death (Feng et al., 2013[14]). Sirt-1 is an NAD^+^-dependent protein deacetylase enzyme that has emerged as a key stress sensor, metabolic sensor, and longevity promoter (Wong and Tang, 2016[53]). Previous studies have reported both *in vitro* and *in vivo* that Sirt-1 expression is significantly decreased in response to Aβ_25-35 _treatment and in an AD model (Lattanzio et al., 2016[22]; Sun et al., 2019[41]). Furthermore, Sirt-1 inhibition promotes excessive cell death in neuronal tissue exposed to Aβ_25-35_. In accordance with previous findings, we also evaluated the role of Sirt-1 in SK-N-SH-treated Aβ_25-35 _cells using EX527, a selective Sirt-1 inhibitor. Inhibition of Sirt1 did not completely decrease apoptosis or GLUT3 impairment in SK-N-SH cells pretreated with Aβ_25-35_. From this result, TMF might partly protect SK-N-SH cells against Aβ_25-35_-induced apoptosis through Sirt1. Consequently, this result suggests that Sirt-1 plays an important role in targeting Aβ_25-35_-induced apoptosis and GLUT3 impairment.

Cellular senescence is widely known as the cellular aging process that occurs before cells undergo dysfunction and apoptosis. Several studies have demonstrated that exposure to toxins, such as hydrogen peroxide (H_2_O_2_), lipopolysaccharide (LPS) or Aβ_25-35_, promotes cellular senescence prior to mediating apoptosis in neuronal tissue (Calvo-Rodríguez et al., 2017[4]; Yin et al., 2016[57]). The upregulation of p21 and p53 together with the decrease in p-Rb/Rb and Sirt1 are closely related to the increase in SA-β-Gal activity, a biomarker of cell aging (Geng et al., 2010[17]; Martire et al., 2013[29]; Zhang et al., 2017[59]). This study demonstrated that the increase in apoptosis and GLUT3 impairment were closely related to the increase in cellular senescence, which was confirmed by upregulation of the number of SA-β-Gal-positive cells, p21, and p53, along with the downregulation of p-Rb/Rb and Sirt1 expression in SK-N-SH cells treated with Aβ_25-35_. Several reports on the effect of flavanone treatment on aging have demonstrated that it potently reduces cellular aging (Da Pozzo et al., 2017[11]; Kean et al., 2015[20]), but TMF's effects are not yet understood. These results demonstrated that TMF treatment significantly reversed cellular aging responses in Aβ_25-35_-treated cells, which might be due to NOX1/4 and Sirt1 mechanisms. On the other hand, a Sirt1 inhibitor progressively increased cellular senescence in response to Aβ_25-35 _treatment. Importantly, cotreatment of TMF with a NOX1/4 and/or Sirt1 inhibitor in Aβ_25-35_ treatment caused a considerably reversed cellular aging response. From these results, TMF protected against Aβ_25-35_-induced senescence in SK-N-SH cells through NOX1/4 and Sirt1.

Nrf2 has been widely studied in the antioxidation pathway, which is upstream of antioxidant enzymes, such as HO-1, SOD, and CAT. Recent studies have reported that Aβ treatment abrogates Nrf2 activation together with decreasing antioxidant enzyme expression (Li et al., 2019[26][27]). In this study, we also found that Aβ_25-35 _treatment in SK-N-SH cells caused a reduction in Nrf2 translocation into the nucleus and reduced HO-1 expression, SOD activity and excessive ROS. In our study, both NOX1/4 and Sirt-1 were found to be targets of the Nrf2 pathway activated by Aβ_25-35_. Several groups have reported that flavonoids and flavanone play a vital role as cellular defense activators by scavenging free radicals and promoting both the Nrf2 and Sirt1 mechanisms (Ali et al., 2018[1]; Velagapudi et al., 2018[47]; Yen et al., 2017[56]). Our data are in line with previous studies showing that TMF significantly attenuates the Aβ_25-35_-suppressed Nrf2 mechanism and antioxidant enzyme expression in neuronal cells mediated by NOX1/4 and Sirt-1. Therefore, NOX1/4 and Sirt-1 are also targets of TMF to protect against cellular defense mechanisms in AD.

Loss of synaptic plasticity is commonly known as the primary event correlated with cognitive impairment in AD, and several studies have documented that the accumulation of Aβ production or toxins in the brain promotes synaptic impairment via several mechanisms, including NOX and Sirt-1 (Gao et al., 2010[16]; Walsh et al., 2014[49]; Zhang et al., 2017[58]). The production of a presynaptic protein (synaptophysin) and a postsynaptic protein (PSD-95) was markedly reduced along with increasing AChE activity in AD (Marsh and Alifragis, 2018[28]). Previous studies have demonstrated the effect of flavonoids and flavanone in neuronal tissue during neurodegeneration and that flavonoids have high potential to reduce excessive intracellular free radicals and proinflammatory cytokines, leading to the promotion of neuronal function in the AD brain (Simunkova et al., 2019[37]). Mechanistic evidence shows that both flavonoids and flavanone can mediate the Nrf2 mechanism together with suppression of NOX, MAPK, and NF-κB mechanisms (Vauzour et al., 2008[46]; Williams and Spencer, 2012[52]), but the role of TMF in synapses remains unclear. In the present study, TMF treatment promoted synaptic protein expression and suppressed AChE activity in response to Aβ_25-35 _treatment. In the case of NOX1/4 and Sirt1 inhibition, TMF progressively promoted neuronal plasticity by upregulating both synaptophysin and PSD-95 and reducing AChE activity in SK-N-SH cells. Therefore, our results suggest that NOX1/4 and Sirt1 might represent downstream targets of the protective role of the TMF in Aβ_25-35_-mediated toxicity.

## Conclusions

**Figure 7:**
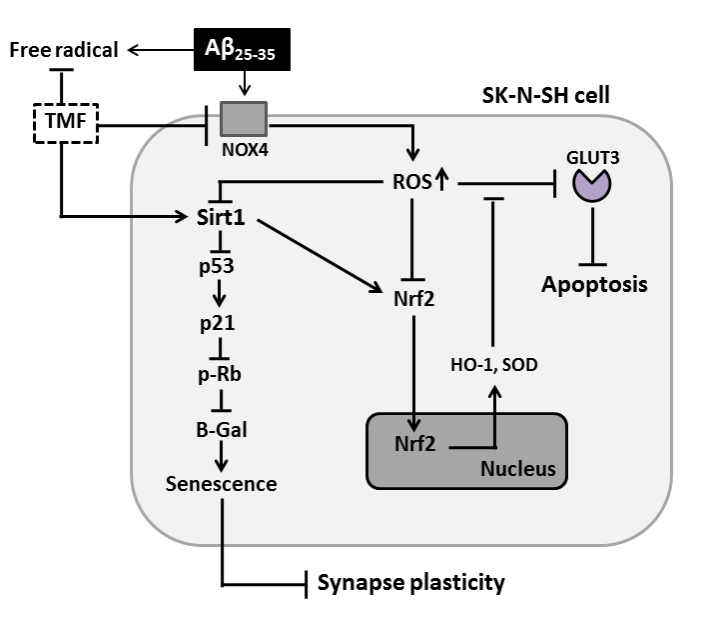
TMF protects against Aβ_25-35_-induced cellular stress, senescence, apoptosis, and impairment of Nrf2 signaling and synaptic plasticity in SK-N-SH cells via NOX4 and Sirt1.

Our study demonstrates that the protective role of TMF in alleviating negative responses, including cell stress response, apoptosis, cell senescence, Nrf2 mechanism impairment and synaptic loss, in Aβ_25-35_-treated SK-N-SH cells was bidirectional in nature, including NOX1/4 inhibition and an activated Sirt1-dependent pathway (Figure 7(Fig. 7)). The present study might have important implications for the use of TMF in AD prevention.

## Acknowledgements

The work was supported by the Faculty of Medicine Research Fund, grant no. 052-2561, Center for Research and Development of Natural Products for Health, Chiang Mai University, Thailand, the Thailand Research Fund (DBG6180030), the Center of Excellence for Innovation in Chemistry, Ministry of Higher Education, Science, Research and Innovation.

## Disclosure of interest

The authors declare no conflicts of interest.
